# [Corrigendum] CXCR6 predicts poor prognosis in gastric cancer and promotes tumor metastasis through epithelial-mesenchymal transition

**DOI:** 10.3892/or.2026.9043

**Published:** 2026-01-05

**Authors:** Jie-Jie Jin, Fa-Xiang Dai, Zi-Wen Long, Hong Cai, Xiao-Wen Liu, Ye Zhou, Qi Hong, Qiong-Zhu Dong, Ya-Nong Wang, Hua Huang

Oncol Rep 37: 3279–3286, 2017; DOI: 10.3892/or.2017.5598

Subsequently to the publication of the above paper, an interested reader drew to the authors' attention that, concerning the cell migration and invasion assay experiments shown in Figs. 3 and 4, the ‘WT/Migration’ and ‘Ctrl/Invasion’ panels in Fig. 3C contained an overlapping section of data, and the ‘WT/Invasion’ and ‘CXCR-sh/Migration’ panels in Fig. 4A were duplicates, such that data which were intended to show the results from differently performed experiments had apparently been derived from the same original sources. Upon examining the data independently in the Editorial Office, it also came to light that the E-cadherin western blot in Fig. 3E was strikingly similar to the N-cadherin western blot shown in Fig. 4A.

However, the authors were able to consult their original data, and recognized that these data had inadvertently been included in these two figures incorrectly. Revised and corrected versions of Figs. 3 and 4, now showing the correct data for the E-cadherin blot in Fig. 3E and the ‘Ctrl/Invasion’ experiment in Fig. 3C, and the ‘CXCR-sh/Migration’ panel in Fig. 4A, are shown on the next page. The authors regret the errors that were made during the compilation of the original figures, and are grateful to the editor of *Oncology Reports* for allowing them the opportunity to publish this Corrigendum. Note that these errors did not have a significant impact on the conclusions reached in this study. All the authors agree with the publication of this corrigendum; furthermore, they apologize to the readership for any inconvenience caused.

## Figures and Tables

**Figure 3.**
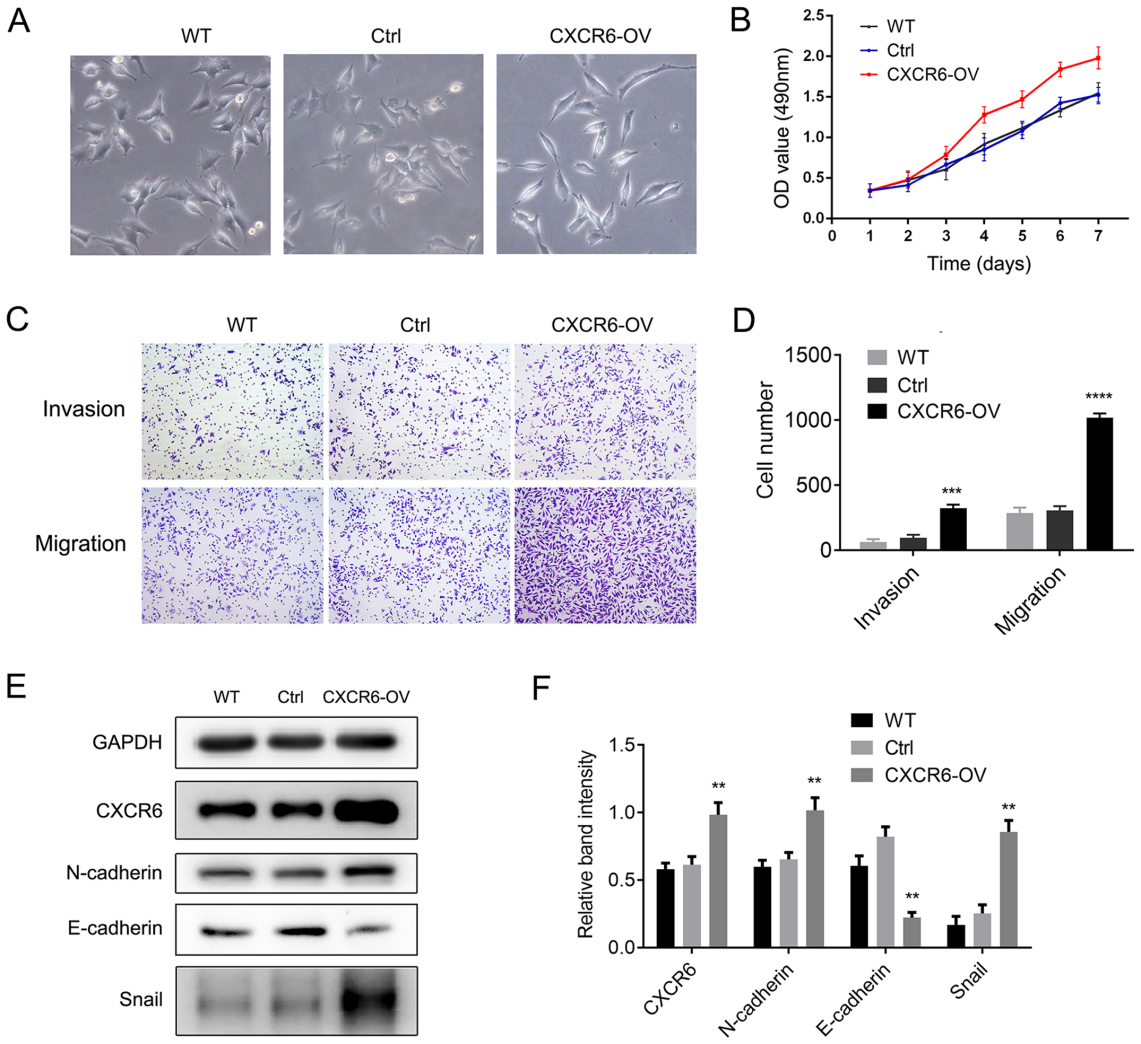
CXCR6 overexpression promotes proliferation, invasion, migration and epithelial-mesenchymal transition (EMT) in gastric cancer (GC) cells. (A) CXCR6 overexpression in HGC27 cells induced the EMT phenomenon with morphological transformation and alterations in cellular configuration in HGC-27 cells. Magnification, ×200. (B) CXCR6 overexpression promoted the proliferation of HGC-27 cells. (C and D) CXCR6 overexpression significantly increased invasion and migration in HGC-27 cells. ***P<0.001, ****P<0.0001. Magnification, ×40. (E and F) CXCR6 overexpression increased N-cadherin and Snail expression, and decreased E-cadherin expression in the HGC-27 cells. **P<0.01. Three independent experiments were conducted.

**Figure 4.**
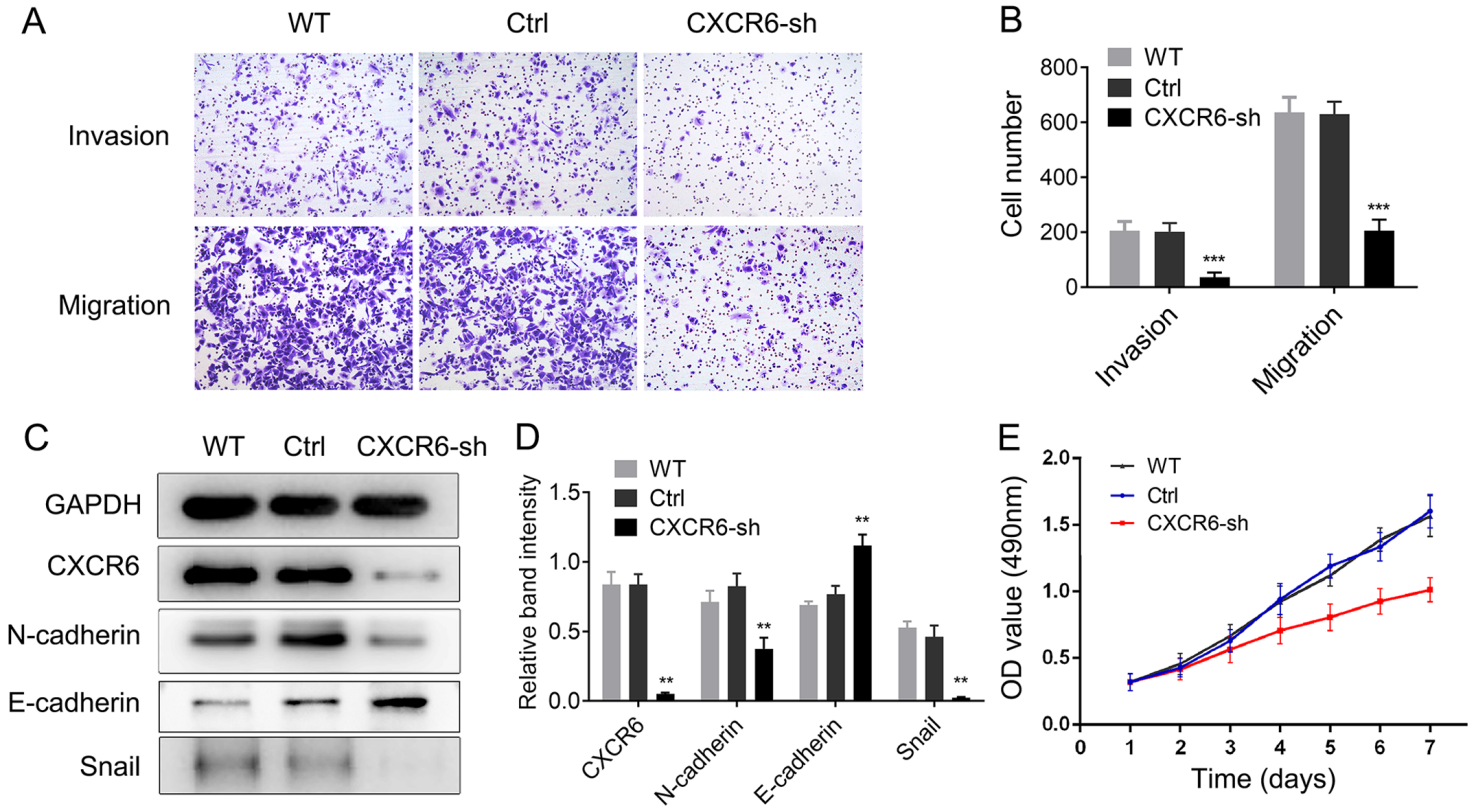
Knockdown of CXCR6 inhibits proliferation, invasion, migration and epithelial-mesenchymal transition (EMT) in gastric cancer (GC) cells. (A and B) CXCR6 knockdown significantly inhibited invasion and migration of the SGC-7901 cells. ***P<0.001. Magnification, ×40. (C and D) CXCR6 knockdown increased E-cadherin expression, decreased N-cadherin and Snail expression in the SGC7901 cells. ***P<0.01. (E) CXCR6 knockdown inhibited cell proliferation in the SGC7901 cells. At least three independent experiments were conducted.

